# Supplementary material for: Those Who Have Continuing Radiation Anxiety Show High Psychological Distress in Cases of High Post-Traumatic Stress: The Fukushima Nuclear Disaster
Source: Int J Environ Res Public Health. 2021 Nov 17;18(22):12048. doi: 10.3390/ijerph182212048 (PMC8623122; doi:10.3390/ijerph182212048)
Supplement: Supplementary file 1 [file ijerph-18-12048-s001.zip › ijerph-1438404-supplementary.pdf]

Health and Information Survey (English-translated version)

January, 2020

1. Which of the following best describes your physical condition in the last month?
Please select one and mark with "○."

1. Extremely good 2. Very good 3. Good 4. Fair 5. Not healthy

2. Regarding your lifestyle,
(1) How many times a month do you exercise or play sports on average? Please select one and mark with "○."

1. Never 2. 1 to 3 times 3. 4 to 7 times 4. 8 to 15 times 5. More than 15 times

(2) Are you satisfied with the quality of your sleep (regardless of length) in the past month? Please select the most appropriate response and mark with "○."

1. Satisfied 2. Slightly dissatisfied 3. Very dissatisfied 4. Very dissatisfied (I could not sleep at all)

(3) Do you drink alcohol every day? Please select one and mark with "○." (*Two small cans for beer, one *go* (180 mL) for sake, 0.7 *go* for shochu, and two small cups or more for whiskey/wine)

1. Yes 2. No 3. I used to drink, but I quit

(4) Do you currently smoke cigarettes almost every day? Please select one and mark with "○."

1. Yes 2. No 3. I used to smoke, but I quit

3. Regarding radiation anxiety,
(1) Just after the Tokyo Electric Power Fukushima Daiichi Nuclear Power Plant accident (hereinafter, nuclear accident), how anxious did you feel about the impact of radiation on your health? Please select the most appropriate response and mark with "○."

1. Not at all 2. Only a little 3. Somewhat 4. Very 5. Extremely

(2) How uneasy do you feel about the impact of radiation on your health now? Please select the most appropriate response and mark with "○."

1. Not at all 2. Only a little 3. Somewhat 4. Very 5. Extremely

4. Do you think you can find and use information on diseases and health on your own if you need it? Please read each statement and mark the most appropriate response with "○."

	I do not think so at all	Somewhat disagree	I can not say either	Somewhat agree	Strongly agree
1. You can collect information from various sources such as newspapers, books, and the Internet.	1	2	3	4	5

2. You can pick out information you want from among a lot of information.	1	2	3	4	5
3. You can understand the information and tell people.	1	2	3	4	5
4. You can determine how reliable the information is.	1	2	3	4	5
5. Based on the information, you can decide plans and actions for health improvement.	1	2	3	4	5

5. For each of the following sentences, please put a ○ in the ( ) if you think "it is correct," × if you think "it is not correct," or △ if you are unsure.

1. Once the body receives radiation, it remains in the body........................ ( )
2. According to international standards, as the exposure dose of radiation increases, the higher the probability of dying from cancer................ ( )
3. In the surveys on the health effects of the second and third generation of atomic bomb survivors in Hiroshima and Nagasaki, genetic influences were not found.. ( )
4. Once damaged by radiation, the DNA (the body of the gene) of cells cannot be repaired............... ( )
5. According to government standards for radioactive materials, the radioactivity level of general food items should not exceed 100 Becquerel per kg.. ( )

6. Now, we ask you about the influence of radiation caused by the nuclear accident, what you experienced, and how you feel about it. Please read each statement below and mark the most appropriate response with "○."

	I do not think so at all	Somewh at disagree	Somewh at agree	I strongly think so
1. I am worried I might suffer from serious diseases due to the influence of radiation in the future.	1	2	3	4
2. Every time my condition gets worse, I become anxious about radiation exposure.	1	2	3	4
3. I am worried that the influence of radiation will be inherited to the next generation, such as my children and grandchildren.	1	2	3	4
4. Looking at reports on nuclear power plant accidents, I become very anxious.	1	2	3	4
5. Because I lived in an area with supposedly high radiation doses, I am worried for myself as well as my children that we might be discriminated against (e.g., receive unfair treatment).	1	2	3	4
6. I try not to talk to people as much as possible about being a local resident of the area.	1	2	3	4
7. I have experienced conflicting opinions with my family about the effects of radiation on health.	1	2	3	4

7. Are you attentive to food radiation and production area?

1. Not at all	2. I used to, but I quit	3. Yes

8. Do you feel you can trust information on radiation based on the source of information (e.g., organization, group, or people)? Please choose 3 sources from the below list that you would trust and mark with "○."

1. International organizations
2. Experts from universities, academic institutions, and others
3. Government ministries
4. Local newspapers (Fukushima Minpo and Fukushima Minyu)
5. National newspapers (Yomiuri, Asahi, Mainichi, and others)
6. NHK
7. Private local broadcast television (FTV, FCT, KFB, TUF)
8. Private national broadcast television
9. Local government
10 Private volunteer groups such as NGOs
11. None of the above

9. What are your sources of information on radiation? Please choose 3 out of the following responses and mark with "○."

1. Local newspapers
2. National newspapers
3. NHK television
4. Private local broadcast television
5. Private national broadcast television
6. Radio
7. Internet news (Yahoo, etc.)
8. Internet sites and blogs other than news
9. SNS (Facebook, Twitter, LINE, etc.)
10. Books and magazines
11. Local government publications
12. Word of mouth (friends and acquaintance)
13. None of the above

10. Has the damage caused by the harmful rumor affected your life?

1. No 2. Somewhat 3. Yes

If you answered 2 or 3, please provide specific details.

____

11. For each of the following questions, please fill in "✓" in the applicable check box.

		I do not think so at all	Somewhat disagree	I can not say either	Somewhat agree	I strongly think so
1	Have you ever been able to do something new in your life after the nuclear accident?	□	□	□	□	□
2	Do you think you have to do what you can since it can't be helped that the nuclear accident has happened?	□	□	□	□	□
3	Do you have time to enjoy yourself, such as hobbies?	□	□	□	□	□
4	Do you feel that you are helpful to others through your job(s), housework, or social activities?	□	□	□	□	□

12. The following questions ask about how you have been feeling during the past 30 days. For each question, please circle the number that best describes how often you had this feeling.

	During the past 30 days, about how often did you feel …	None of the time	A little of the time	Some of the time	Most of the time	All the time
1	…nervous?	0	1	2	3	4
2	…hopeless?	0	1	2	3	4
3	…restless or fidgety?	0	1	2	3	4
4	…so depressed that nothing could cheer you up?	0	1	2	3	4
5	…that everything was an effort?	0	1	2	3	4
6	…worthless?	0	1	2	3	4

13. We would like to ask you about your experience of the Great East Japan Earthquake. Below is a list of problems and complaints that veterans sometimes have in response to stressful life experiences. Please read each one carefully, circle in each number to indicate how much you have been bothered by that problem in the last month.

		Not at all	A little bit	Moderately	Quite a bit	Extremely
1	Repeated, disturbing memories, thoughts, or images of a stressful experience from the past?	1	2	3	4	5
2	Having physical reactions (e.g., heart pounding, trouble breathing, or sweating) when something reminded you of a stressful experience from the past?	1	2	3	4	5

3	Avoid activities or situations because they remind you of a stressful experience from the past?	1	2	3	4	5
4	Having difficulty concentrating?	1	2	3	4	5

14. Finally, we ask you about yourself and basic matters concerning your home and family. There are questions related to your private life, but they are necessary in order to obtain accurate results. We thank you in advance for your contribution.

1） Please tell us your sex and age.

1. Male 2. Female （ ） years

2) Which of the following best describes your current family status? Please select one and mark with "○."

1. Single household (only yourself) 2. Couple-only household 3. Couple and unmarried children household
4. Household of unmarried children and you 5. Third generation family 6. Other

3） What is the highest educational qualification that you have completed? Please select one and mark with "○."

1. Junior high school 2. High school 3. Junior college/vocational school 4. University/graduate school

4） Which of the following is your current residence? Please select one and mark with "○."

1. Owned house 2. Rented house or apartment 3. Temporary housing 4. Government subsidized housing
5. Public housing 6. Home of friend/relative 7. Other

5） Which of the following was your residence before the disaster? Please select one and mark with "○."

1. Owned house 2. Rented house or apartment 3. Home of friend/relative 4. Other ( )

6） Have you and your family moved from your original address to avoid radiation?

1. Yes 2. We moved for other reasons 3. No

If you selected 3, please skip 7).

7) If you selected 1 or 2 in 6), please choose one of the following responses and mark with "○."

1. I and my family moved together 2. Only I moved 3. Only some family members moved
4. My family members and/or I evacuated immediately after the earthquake but quickly returned

8） At the time of the Great East Japan Earthquake, did your family have a child/ren or pregnant woman? Please circle all that apply.

1. We had a child(ren) under age 18 2. We had a child(ren) over age 19
3. (Female only) I was pregnant 4. We had a pregnant woman 5. None

9) Are you currently working? Please select one and mark with "○." (Even if you are a househusband or housewife, if you are currently working part-time, etc., please select "Working.")

1. Working (include self-employed and part-time workers)
2. I am on leave
3. Not working (student, househusband/wife, job seeker)

10) How do you think about people in your area you live in? Please read each statement and mark the most appropriate response with "○".

	I do not think so at all	Somewhat disagree	I can not say either	Somewhat agree	Strongly agree
1. People living in the area help each other.	1	2	3	4	5
2. I can trust people living in the area.	1	2	3	4	5
3. People living in the area greet each other.	1	2	3	4	5
4. If problems occur in the area, people work together to try to resolve the problems.	1	2	3	4	5

11) Are you enrolled in the following organizations or groups? Please mark as many responses as appropriate with "○."

1. Neighborhood association · resident association
2. Regional groups such as youth group, women's association, elderly association, PTA, child association (training group)
3. NPO, volunteer/citizen activity organization, co-operative association
4. Vocational organizations such as business association, peer association, industry group, labor union, etc.
5. Other（　　　　　　　　　　　　　　　　　　）
6. No

◇ Please provide any additional comments below.

Thank you for your cooperation.

健康と情報についての調査

2020 年１月

１．最近１か月間のあなたの身体的な健康状態は次のどれにあたりますか。最もあてはまるもの１つに〇を付けてください。

１　きわめて良い	２　とても良い	３　良い
４　まあまあ	５　不健康	

２．あなたの生活習慣について

（１）汗がでるくらいの運動やスポーツを、１カ月に平均何回くらいしましたか。１つ選んで〇を付けてください。

１　していない	２　１～３回	３　４～７回	４　８～15 回	５　15 回より多い

（２）あなたは、ここ１か月間、（睡眠の長さに関わらず）睡眠の質に満足していますか。最もあてはまるもの１つ選んで〇を付けてください。

１　満足している	２　少し不満	３　かなり不満	４　非常に不満か、全く眠れなかった

（３）あなたは、お酒・アルコール*を毎日飲んでいますか。１つ選んで〇を付けてください。（*ビールなら小缶で２本、日本酒なら１合、焼酎なら 0.７合、ウイスキー・ワインなら小グラス２杯、以上）

１　はい	２　いいえ	３　以前飲んでいたが、やめた

（４）現在、ほぼ毎日、たばこを吸っていますか。１つ選んで〇を付けてください。

１　吸っている	２　吸っていない	３　以前吸っていたが、やめた

３．放射線に関する不安について

（１）東京電力福島第一原子力発電所の事故（以下、原発事故とします）が起きた直後、あなたの健康への放射線の影響について、どのくらい不安を感じましたか。最もあてはまるもの１つに〇を付けてください。

１　全くない	２　少ししかない	３　いくらか	４　たくさん	５　非常に

（２）現在、あなたの健康への放射線の影響について、どのくらい不安を感じますか。最もあてはまるもの１つに〇を付けてください。

１　全くない	２　少ししかない	３　いくらか	４　たくさん	５　非常に

4．もし必要になったら、病気や健康に関連した情報を、自分自身で探したり利用したりすることができると思いますか。それぞれの文章を読んで、最もあてはまるもの１つに〇を付けてください。

	全くそう思わない	どちらかといえばそう思わない	どちらとも言えない	どちらかといえばそう思う	強くそう思う
1　新聞、本、インターネットなど、色々な情報源から情報を集められる。	1	2	3	4	5
2　たくさんある情報の中から、自分の求める情報を選び出せる。	1	2	3	4	5
3　情報を理解し、人に伝えることができる。	1	2	3	4	5
4　情報がどの程度信頼できるかを判断できる。	1	2	3	4	5
5　情報をもとに健康改善のための計画や行動を決めることができる。	1	2	3	4	5

5．以下の文章で「正しいと思う」ものに〇を、「正しくないと思う」ものに×を、どちらか「分からない」ものには△を（　）の中にご記入下さい。

1	放射線を一度身体に受けるとその放射線はずっと体内に残る。……………………………（　）
2	国際的な基準では、放射線の被ばく量が多いほど、そのためにガンで死亡する確率も高くなるという考え方が採用されている。………………………………………………………………（　）
3	広島、長崎の原爆被ばく者の二世、三世の健康影響に関する調査では、遺伝的影響は認められていない。……………………………………………………………………………………（　）
4	放射線でいったん傷ついた細胞の DNA（遺伝子の本体）は修復することができない。…（　）
5	政府による放射性物質の基準値では一般食品は 1 kg あたり 100 ベクレルを超えないように設定されている。………………………………………………………………………………（　）

6．原発事故による放射線の影響について、感じていることや、経験されたことについて伺います。それぞれの文章を読んで、最もあてはまるもの１つに〇を付けてください。

	全くそう思わない	あまりそう思わない	ややそう思う	とてもそう思う
1　将来、放射線の影響で深刻な病気にかかるのではないかと心配している。	1	2	3	4
2　体の具合が悪くなるたびに、放射線を浴びたせいではないかと不安になる。	1	2	3	4
3　放射線の影響が子どもや孫など次の世代に遺伝するのではないかと心配している。	1	2	3	4
4　原発事故に関する報道を見ると、とても不安になる。	1	2	3	4
5　放射線量が高いといわれる地域に住んでいたために、自分や子どもが他の人から差別される（不公平な扱いを受ける）不安がある。	1	2	3	4
6　その地域の住民であることを、なるべく人に話さないようにしている。	1	2	3	4
7　放射線が健康に与える影響について、家族と意見が対立して、もめた経験がある。	1	2	3	4

７．あなたは、原発事故の発生後から現在までに、食べ物の放射線量と産地に気をつけていますか。

１．していない	２．以前はしていたが、今はしていない	３．している

８．放射線について、その報道の元となる情報が、どこからもたらされたものならば信用できると思いますか? おもなものを３つ選び、○をつけてください。

1	国際機関（国連　WHO など）などが発表した情報
2	大学・研究所等の専門家が発表した情報
3	政府・省庁が発表した情報
4	地元新聞（福島民報、福島民友）が発表した情報
5	全国新聞（讀賣新聞、朝日新聞、毎日新聞等）が発表した情報
6	NHK が発表した情報
7	地元民放テレビ（FTV、FCT、KFB、TUF）が発表した情報
8	全国民放テレビ（フジ、日本テレビ、テレビ朝日、TBS 等）が発表した情報
9	地方自治体が発表した情報
10	NGO や市民団体、民間のボランティア団体等が発表した情報
11	この中にはない

９．ふだんあなたは、放射線に関する情報をどこから得ていますか。以下のうちから、おもなものを３つ選び、○をつけてください。

1	地元新聞（福島民報、福島民友）
2	全国新聞（讀賣新聞、朝日新聞、毎日新聞等）
3	NHK テレビのニュース、番組
4	地元民放テレビ（FTV、FCT、KFB、TUF）制作のニュース・番組
5	全国民放テレビ（フジ、日本テレビ、テレビ朝日、TBS 等）制作のニュース・番組
6	ラジオ
7	インターネットのニュース（Yahoo ニュース等）
8	インターネットのニュース以外のサイト・ブログ
9	SNS（フェイスブック、ツイッター、LINE 等）
10	雑誌・書籍
11	自治体公報（県公報も含む）
12	クチコミ（知人、友人等）
13	その他

１０．原発事故に伴う風評被害は、暮らしに影響しましたか？

１　なし　　２　多少あり　　３　あり

２、３と答えた方、具体的にお書きください。

____

１１．次のそれぞれの質問について、当てはまる□に✓をご記入ください。

		全く当てはまらない	あまり当てはまらない	どちらともいえない	やや当てはまる	かなり当てはまる
1	災害後の生活の中で新たにできるようになったことがありますか。	□	□	□	□	□
2	災害は仕方がないので、自分のできることをやるしかないと思いますか。	□	□	□	□	□
3	趣味など自分が楽しむ時間をもつことができていますか。	□	□	□	□	□
4	自分の仕事や家事、社会活動を通して、人の役に立っていると感じていますか。	□	□	□	□	□

１２．過去 30 日の間に、どれくらいの頻度で次のことがありましたか。当てはまる数字を○で囲んでください。

		全くない	少しだけ	ときどき	たいてい	いつも
1	神経過敏に感じましたか。	0	1	2	3	4
2	絶望的だと感じましたか。	0	1	2	3	4
3	そわそわ、落ち着かなく感じましたか。	0	1	2	3	4
4	気分が落ち込んで、何が起こっても気が晴れないように感じましたか。	0	1	2	3	4
5	何をするにも骨折りだと感じましたか。	0	1	2	3	4
6	自分は価値のない人間だと感じましたか。	0	1	2	3	4

１３．東日本大震災の体験についてお尋ねします。以下の質問は、人々が人生におけるストレスの多い経験（以下「ストレス体験」という）をした際、その経験に対して時々起こる問題や訴えのリストです。各項目をよく読んで、この１か月の間、その問題にどのくらい悩まされていたかについて、当てはまる数字を○で囲んでください。

		全くなかった	少しあった	中程度であった	かなりあった	非常にあった
1	そのストレス体験の、心をかき乱すような記憶、考え、イメージ（光景など）を繰り返し思い出す。	1	2	3	4	5
2	何かのきっかけでそのストレス体験を思い出したとき、身体が反応する。（例：心臓がドキドキバクバクする、息苦しくなる、汗ばむ）	1	2	3	4	5
3	そのストレス体験を思い出させられるため、特定の活動や状況を避ける。	1	2	3	4	5
4	物事に集中できない。	1	2	3	4	5

１４．最後に、あなたご自身、あるいはご家庭の基本的なことがらについて伺います。立ち入った質問もありますが、正確な結果を出すために伺うことが必要です。よろしくお願いします。

１）あなたの性別・年齢を教えてください。

１　男　性	２　女　性	（　　　　　　）歳

２）現在、あなたと同居している家族の構成は、つぎのどれにあたりますか。１つ選んで○を付けてください。

１　単身世帯（自分のみ）	２　夫婦のみ	３　夫婦と未婚の子の世帯
４　あなたと未婚の子の世帯	５　三世代家族	６　その他（　　　　　　）

３）あなたの最終学歴は次のどれですか。１つ選んで○を付けてください。

１　中学まで	２　高校	３　短大・専門学校	４　大学・大学院

４）現在のお住まいは次のどれにあたりますか。１つ選んで○をつけてください。

１　自宅	２　借家や賃貸アパート	３　仮設住宅	４　借り上げ住宅
５　公営住宅	６　知人・親戚の家	７その他（　　　　　　）	

５）震災前のお住まいは次のどれにあたりますか。１つ選んで○をつけてください。

１　自宅	２　借家や賃貸アパート	３　知人・親戚の家
４　その他　（　　　　　　）		

６）あなたとあなたの家族は、放射線を避けるために、もとの住所地から転居されましたか。

１　転居した ↓	２　別の理由で転居した	３　転居しなかった ──→ ７）は飛ばしてください

７）６）で「転居した」と答えた方は、１つ選んで○をつけてください。

１　自分および家族がともに転居した	２　自分のみ転居した
３　家族のみ転居した	４　自分または家族が震災直後に一時的に避難した

８）東日本大震災のとき、ご家族にはお子さん、または、妊婦さんがいましたか。
該当するもの全てに○をつけてください。

１　18 歳以下の子供がいた	２　19 歳以上の子供がいた	
３（女性のみ）自分が妊娠していた	４　家族が妊娠していた	５　いずれでもない

９）あなたは現在お仕事をしていますか。なお主夫・主婦の方でも、現在パートなどでお勤めの場合は「働いている」とお答えください。１つ選んで〇をつけてください。

１　働いている（勤め・自営・パートを問いません）

２　休職中である

３　働いていない（学生、専業主夫・主婦、求職中を含みます）

１０）あなたの今住んでいる地域の人々についてどう感じていますか。それぞれの文章を読んで、最もあてはまるものに〇を付けてください。

	全くそう思わない	どちらかと言えばそう思わない	どちらとも言えない	どちらかと言えばそう思う	強くそう思う
１　今住んでいる地域の人々はお互いに助け合っている。	1	2	3	4	5
２　今住んでいる地域の人々は信頼できる。	1	2	3	4	5
３　今住んでいる地域の人々はお互いにあいさつをしている。	1	2	3	4	5
４　今住んでいる地域で問題が生じた場合、人々は力を合わせて解決しようとする。	1	2	3	4	5

１１）あなたは、つぎにあげる組織や団体に加入していますか。加入しているものにいくつでも〇をつけてください。

1　町内会・自治会

2　青年団・婦人会・老人会、ＰＴＡ、子ども会(育成会)などの地域団体

3　ＮＰＯ、ボランティア・市民活動団体、生活協同組合などの任意団体

4　商店会・同業組合・業界団体、労働組合などの職業団体

5　その他（　　　　　　　　　　　　　　　　　　　　　　）

6　とくに加入していない

◇　全体を通じて、何でも結構ですので、お書き頂ければ幸いです。

このたびは、調査にご協力頂き、ありがとうございました。
